# Effects of physical activity on executive function and its subdomains in children aged 5–6

**DOI:** 10.3389/fpsyg.2025.1651806

**Published:** 2025-12-17

**Authors:** Baolong Wang, Peiyou Chen, Zhihao Jia, Zhaowen Tan

**Affiliations:** 1 College of Sports Science, Nanjing Normal University, Nanjing, China; 2 College of Physical Education, Nanjing Xiaozhuang University, Nanjing, China

**Keywords:** physical activity, executive function, children aged 5–6, inhibitory control, cognitive flexibility, working memory

## Abstract

**Objective:**

The purpose of this study is to explore the effect of physical activity on the executive function of 5–6-year-old children and to provide a theoretical and empirical basis for further research on improvements in the executive function of children caused by physical activity.

**Methods:**

A total of 170 children (5–6 years old) from several kindergartens were selected via multistage stratified sampling. All the children wore 7-day accelerometers (ActiGraph GT3X) to measure their daily physical activities. Parents completed the preschool children’s executive function questionnaire (BRIEF-P) to assess their daily executive function.

**Results:**

(1) The total duration of physical activity (TPA) was 110.84 ± 22.52 min/day, the duration of low-intensity physical activity (LPA) was 36.23 ± 7.53 min/day, and the duration of medium- and high-intensity physical activity (MVPA) was 74.55 ± 16.77 min/day. A total of 82.6% of the children reached the recommended amount of MVPA. (2) After adjusting for body mass index (BMI), parents’ highest educational background and parents’ total monthly income, MVPA was negatively correlated with children’s total executive function score (*β* = −0.217, *p* = 0.007, 95% CI: −0.374 to −0.060, *R*^2^ = 0.034), inhibition function score (*β* = −0.103, *p* = 0.003, 95% CI: −0.170 to −0.036, *R*^2^ = 0.062), cognitive flexibility score (*β* = −0.079, *p* = 0.006, 95% CI: −0.135 to −0.023, *R*^2^ = 0.038) and working memory score (*β* = −0.090, *p* = 0.015, 95% CI: −0.163 to −0.018, *R*^2^ = 0.018). (3) There was an inverted U-shaped relationship between MVPA and children’s total executive function and working memory performance. An MVPA of 71.39 to 83.33 min/day had the greatest effect on children’s total executive function and working memory. (4) Compared with girls, boys’ MVPA time per day increased, and their total executive function, inhibitory function and working memory improved. Boys’ TPA time per day increased, and their cognitive flexibility improved.

**Conclusion:**

Physical activity can improve the executive function of children aged 5–6 years to some extent. MVPA can improve children’s executive function and subdomains, and there is a correlation between boys’ physical activity and executive function.

## 1 Background

With the widespread use of electronic devices, screen time and sedentary behavior among young children have increased, leading to higher rates of myopia and obesity, as well as reduced levels of physical activity (Pate et al., 2015). Insufficient physical activity not only adversely affects the body composition and physical development of young children but also hinders the development of their behavioral qualities and social behaviors (Clark et al., 2020).

Early childhood, characterized by strong brain plasticity and neurogenesis, is a critical period for the development of cognitive, social, and emotional abilities. Executive function (EF), a high-level cognitive process, plays a vital role in shaping children’s brain development, psychology, language, memory, and other abilities (Mora-Gonzalez et al., 2019). Executive function generally consists of three interrelated subdomains: inhibitory control, cognitive flexibility, and working memory (Hillman and Biggan, 2017; Tomporowski et al., 2008). Inhibitory control refers to the ability to suppress impulsive responses and focus attention on goal-directed behavior; cognitive flexibility is the capacity to shift between tasks or mental sets and adapt to changing environmental demands; and working memory involves maintaining and manipulating information over short periods. These core components jointly support self-regulation, problem solving, and adaptive behavior in preschoolers, forming the foundation for early academic and socioemotional development (Donnelly et al., 2016; van der Fels et al., 2015). Recent studies have further demonstrated the close link between physical activity and EF development in preschool-aged children. For instance, structured activities such as mini-trampoline training significantly improve executive and motor functions in 4–6-year-olds (Ltifi et al., 2025). Similarly, ecological studies have shown that daily behaviors, including physical activity and sleep, are associated with executive and developmental outcomes in early childhood (Ltifi et al., 2024a; Ltifi et al., 2024c). These findings provide experimental and cross-cultural evidence supporting the critical role of physical activity in early EF development. Therefore, focusing on the development of executive function in early childhood may promote long-term benefits, and physical activity is an important influencing factor.

In recent years, the role of physical activity in supporting cognitive and neurodevelopmental health has gained increasing attention (Donnelly et al., 2016; van der Fels et al., 2015). Regular physical activity enhances children’s physical fitness and cognitive abilities while fostering long-term health habits across the lifespan (Puder et al., 2011; Zeng et al., 2017; Álvarez-Bueno et al., 2017; Hancox and Rasmussen, 2018). Previous research has mainly examined adolescents and adults, showing that moderate-to-vigorous physical activity improves inhibitory control and working memory (Fanning et al., 2017). However, findings among preschool-aged children remain inconsistent, possibly due to differences in exercise type, duration, or intensity (Álvarez-Bueno et al., 2017; Chang and Wang, 2020).

Neurophysiological models suggest an inverted U-shaped relationship between exercise intensity and executive function, indicating that moderate-intensity activity may most effectively activate the prefrontal cortex and enhance cognitive performance (Quan et al., 2020; Jiang and Zeng, 2015). Physical activity may influence different executive function (EF) subdomains in distinct ways. Acute activity can temporarily boost EF, whereas consistent engagement produces more stable benefits. Yet, most existing studies have focused on school-aged children, adolescents, and adults, leaving preschoolers, especially those aged 5–6 years, relatively underexplored (Hillman and Biggan, 2017; Wang and Tian, 2018; Kong et al., 2019; Alesi et al., 2016; Sun, 2017). Some studies have found that moderate to high intensity activity improves sustained attention and behavioral inhibition in preschoolers (Fanning et al., 2017; Palmer et al., 2013), while others reported no significant associations between activity levels and EF (St Laurent et al., 2023). Such discrepancies may arise from methodological differences in assessing both physical activity and EF, including the use of subjective versus objective measures.

Age also serves as an important moderating factor in the relationship between physical activity and executive function. Neurological maturation processes such as synaptic pruning, myelination, and prefrontal cortex development differ across ages, influencing EF performance. In addition, hormonal variations such as changes in cortisol and growth-related hormones may alter physiological responses to exercise, leading to age-specific effects of physical activity on EF (Tomporowski et al., 2008). Understanding these mechanisms is essential for identifying optimal activity levels for children at different developmental stages. Furthermore, research indicates that gender differences may exist in young children’s physical activity behaviors, with boys typically engaging in higher-intensity activities and more vigorous play than girls (Hoffmann and Powlishta, 2001). Evidence further suggests that gender may moderate the relationship between physical activity and executive function. Some studies have identified this association primarily among boys (Cook et al., 2019), while others indicate a stronger link between physical activity and cognitive outcomes in girls (Killgore and Schwab, 2012). These inconsistent findings suggest that gender may influence the pattern of association between physical activity and different subdomains of executive function.

In China, research on preschoolers’ physical activity remains limited, particularly studies using objective measurement tools. Few investigations have explored the relationship between physical activity and executive function among children aged 5–6 years. Therefore, this study aimed to objectively examine the relationship between physical activity and executive function in 5–6-year-old children using accelerometer data and standardized EF assessments, providing theoretical and empirical evidence to support interventions that enhance EF through physical activity. Additionally, by incorporating gender as a variable, the study offers theoretical grounding for identifying potential gender differences in young children’s executive function.

## 2 Materials and methods

### 2.1 Research subjects

A multistage hierarchical sampling method was used to select 5–6-year-old children from three public kindergartens in Langya District, Chuzhou City, Anhui Province, China (permanent resident population: 272,000; registered population: 281,700; urbanisation rate: 97.78%, classified as urban rather than rural). Kindergartens were stratified by size and geographic location, and classes were randomly selected within each kindergarten. Inclusion criteria were: (1) children aged 5–6 years attending full-day kindergarten; (2) no diagnosed neurological, developmental, or musculoskeletal disorders; and (3) written informed consent obtained from parents or guardians. Exclusion criteria included: (1) chronic illness limiting physical activity; (2) inability to wear the accelerometer continuously for seven days. A total of 170 children (85 boys and 85 girls) were recruited to participate in the test. After data cleaning, physical activity and executive function data were complete and valid for 149 children, with an overall validity rate of 87.6%, including 70 boys and 79 girls (Table 1). Data from 21 children were excluded due to incomplete questionnaire data (*n* = 9), insufficient accelerometer wear time (<3 valid days, *n* = 8), and device malfunction (*n* = 4). A sensitivity analysis comparing demographic characteristics between included and excluded participants revealed no significant differences, confirming the absence of sampling bias. The study protocol and informed consent procedure were approved by the Ethics Committee in Research of Human Subjects at Nanjing Normal University (code: 202304005).

**Table 1:** List of subjects’ basic information.

Basic information	Boy (*n* = 70)	Girl (*n* = 79)	Total (*n* = 149)
Height (cm)	117.23 ± 0.05	116.30 ± 0.05	116.73 ± 0.05
Weight (kg)	22.06 ± 3.74	21.03 ± 3.46	21.52 ± 3.62
BMI (kg/m^2^)	15.96 ± 1.77	15.48 ± 1.82	15.71 ± 1.81
BMI rating (%)			
Normal (*n* = 115)	78.5	81.0	79.9
Overweight (*n* = 19)	8.6	6.3	7.4
Obese (*n* = 15)	12.9	12.7	12.8
Average gross monthly income of parents (¥) (%)			
1,000–5,000 (*n* = 44)	35.7	24.1	29.5
5,001–10,000 (*n* = 78)	52.9	51.9	52.3
>10,000 (*n* = 27)	11.4	24.1	18.1
Highest parental education (%)			
Middle school and below (*n* = 48)	31.4	32.9	32.2
Senior middle school (*n* = 52)	35.7	36.7	36.2
University and above (*n* = 47)	32.9	30.4	31.6

BMI, body mass index.

### 2.2 Research methods

#### 2.2.1 Literature method

We searched for “preschoolers,” “physical activity,” “executive function,” and related combined keywords (e.g., “children AND executive function,” “physical activity AND cognition”) using CNKI, Web of Science, Science Direct, PubMed, and other databases. The search covered studies published between January 2015 and March 2024, both in Chinese and English. A total of 87 relevant articles were retrieved. Inclusion criteria were: (1) empirical studies involving preschool children aged 3–6 years; (2) studies that objectively or subjectively measured physical activity and executive function; and (3) peer-reviewed journal publications. These studies were analyzed, compared, and summarized to provide a theoretical and empirical basis for this study.

#### 2.2.2 Questionnaire method

A questionnaire was completed by the subjects’ parents to obtain basic information about the subjects, including sex, age, height, weight, highest parental education, and average gross monthly parental income. The parental questionnaire was a standardized tool previously published and validated in preschool population studies (Quan et al., 2020), and was used in this study without modification to ensure methodological consistency. Among these, the Body Mass Index (BMI) is calculated for preschool children using the formula: BMI = weight (kg) / height (m)^2^. This serves to determine the presence of obesity, with the standard BMI range being 15–18. A BMI exceeding 25 indicates overweight, while a BMI below 15 signifies underweight, potentially indicating malnutrition (Quan et al., 2020).

#### 2.2.3 Measurement methods

##### 2.2.3.1 Physical activity measurement

An ActiGraph GT3X triaxial accelerometer was used to measure physical activity in young children. The subjects were instructed to wear the accelerometer on the upper right iliac crest at all times except for bathing, swimming and sleeping, and for 7 consecutive days (including 5 weekdays and 2 weekend days). To ensure compliance, parents were asked to record daily wear time in a simple logbook and were contacted by phone every 2 days to confirm proper wearing status. The staff collected the devices on the eighth day after testing and processed the data using ActiLife software (version 6.13.2) (Quan et al., 2020). (See Table 2 for the accelerometer parameter settings for the physical activity measurements).

**Table 2:** Physical activity measurement parameter settings for accelerometers.

Serial number	Parameter content	Parameter setting
1	Testing instruments	ActiGraph GT3X
2	Acquisition interval	1 s
3	Definition of unworn time	Choi arithmetic
4	How many hours of wear per day is the data valid for that day	≥480 min
5	At least a few days of valid data for statistical analysis	Minimum 3 days, 2 working days + 1 weekend day
6	Different intensity thresholds	Pate
	SB	Counts ≤ 799 / min
	LPA	799 < Counts ≤ 1,679 / min
	MPA	1,680 < Counts ≤ 3,367 / min
	VPA	Counts ≥ 3,368 / min

SB, sedentary behavior; LPA, low-intensity physical activity time; MPA, moderate-intensity physical activity time; VPA, vigorous-intensity physical activity time.

##### 2.2.3.2 Measurement of executive function

In this study, the Behavior Rating Scale of Executive Function-Preschool Version (BRIEF-P) was used to assess preschool children. The questionnaire consists of 63 items divided into 5 factors and 3 dimensions. The 5 factors include inhibition, switching, emotional control, working memory, and organizational factors. The inhibition and affective control factors constitute the self-regulation index, the conversion and affective control factors constitute the cognitive flexibility index, and working memory and organizational planning constitute the working memory index. A higher score indicates a higher level of executive dysfunction, reflecting poorer performance in the evaluated domains (Quan et al., 2020). The Chinese version of the BRIEF-P has been psychometrically validated in local preschool populations, demonstrating good internal reliability, with the Cronbach’s *α* coefficient reported as 0.89 in previous studies. In this study, the internal consistency of the BRIEF-P was also verified (Cronbach’s α coefficient = 0.90).

#### 2.2.4 Statistical analysis

This study utilized SPSS 22.0 software to statistically process the data, which are presented as the means ± standard deviations (M ± SDs) for data that conformed to a normal distribution and medians and interquartile ranges for data that did not conform to a normal distribution. The Shapiro–Wilk test was used to examine the normality of each continuous variable before analysis. Comparisons between groups that met the normal distribution criteria were analyzed via ANOVA, and comparisons between groups that did not meet the normal distribution criteria were performed via the chi-square test. Before the data were analyzed, a linear regression model was first used to diagnose multicollinearity, and the independent variables were adjusted according to the diagnostic results. In the regression model, children’s executive function scores were treated as the dependent variable, and physical activity indicators (LPA, MPA, VPA, MVPA) were included as independent variables, with covariates such as BMI, parental education, and parental income controlled. A linear regression model was subsequently used to explore the effect of physical activity on improving executive function in 5–6-year-olds. The level of statistical significance was set at *p* < 0.05.

## 3 Results

### 3.1 Physical activity level of young children

During the 7 days that the subjects wore the accelerometer to measure physical activity, the average wearing time per day was 722.69 ± 45.39 min. The results of the physical activity levels of the young children revealed that LPA was correlated with overweight and obese young children (*r* = 0.267, 95% CI: 0.082 to 0.431, *p* = 0.014), while total physical activity time (TPA) was correlated specifically with overweight children (*r* = 0.281, 95% CI: 0.097 to 0.443, *p* = 0.010). SBs were correlated with young children whose parents’ average monthly gross income was greater than 10,000 (*r* = 0.214, 95% CI: 0.038 to 0.376, *p* = 0.018), and wearing time was correlated with young children whose parents’ average monthly gross income was 5,001–10,000 (*r* = 0.314, 95% CI: 0.153 to 0.458, *p* = 0.002). LPA and wearing time were correlated (*r* = 0.243, 95% CI: 0.074 to 0.398, *p* = 0.011) with young children whose parents’ highest education level was university or higher (Table 3).

**Table 3:** Table of physical activity level of young children (min/day).

Classification	SB	LPA	MVPA	TPA	Wearing time
Sex					
Boy (*n* = 70)	616.09 ± 41.01	36.93 ± 7.77	75.22 ± 16.65	112.15 ± 22.47	715.10 ± 42.40
Girl (*n* = 79)	608.46 ± 42.65	35.60 ± 7.29	73.95 ± 16.96	109.68 ± 22.64	608.46 ± 42.66
BMI rating					
Normal (*n* = 115)	613.69 ± 42.97	35.38 ± 6.90	73.18 ± 16.10	108.64 ± 21.16	724.14 ± 47.05
Overweight (*n* = 19)	600.53 ± 42.88	40.05 ± 9.22^#^	83.14 ± 16.58	123.19 ± 24.49 ^#^	730.14 ± 41.21
Obese (*n* = 15)	608.43 ± 34.87	39.32 ± 9.06^#^	78.18 ± 19.70	117.50 ± 26.96	709.27 ± 35.35
Average gross monthly income of parents (¥)					
1,000–5,000 (*n* = 44)	601.37 ± 43.48	36.18 ± 8.33	75.48 ± 17.90	111.88 ± 23.04	706.73 ± 47.30
5,001–10,000 (*n* = 78)	614.54 ± 40.75	36.97 ± 7.97	75.18 ± 16.69	112.15 ± 23.05	731.1 ± 41.82^**^
>10,000 (*n* = 27)	622.23 ± 40.50^*^	34.16 ± 6.55	71.20 ± 15.19	105.36 ± 19.91	724.30 ± 46.92
Highest level of parental education					
Middle school and below (*n* = 48)	604.86 ± 45.27	34.10 ± 7.35	71.17 ± 19.65	105.47 ± 25.39	713.16 ± 43.59
Senior middle school (*n* = 52)	614.68 ± 41.35	36.83 ± 7.48	76.17 ± 15.91	113.01 ± 22.01	721.59 ± 46.17
University and above (*n* = 47)	616.36 ± 38.87	37.71 ± 7.43^&^	76.13 ± 14.18	113.84 ± 19.24	733.6 ± 44.84^&^
Total (*n* = 149)	612.05 ± 41.93	36.23 ± 7.53	74.55 ± 16.77	110.84 ± 22.52	722.69 ± 45.39

LPA and MVPA in BMI subgroups did not and were compared by the chi-square, while other variables were analyzed using one-way ANOVA. Parental monthly income groups (¥1,000–5,000, ¥5,001–10,000, and >¥10,000) corresponded approximately to low, medium, and high levels based on the local minimum wage (¥2,200/month in 2023). VS normal: ^#^*p* < 0.05; VS 1000–5,000: ^*^p < 0.05, ^**^p < 0.01; VS middle school and below: ^&^*p* < 0.05.

According to the recommended standards of the Exercise Guidelines for Preschoolers (3–6 years old), which were first developed for Chinese preschoolers in 2020, preschoolers should accumulate more than 60 min of MVPA per day (Wang and Tian, 2018). The MVPA rates of young children of different genders revealed that 82.6% of them reached the recommended amount of MVPA (Table 4). Although boys (75.22 ± 16.65 min/day) had slightly higher MVPA than girls (73.95 ± 16.96 min/day), the difference was not statistically significant (*t* = 0.484, 95% CI: −4.031 to 6.571, *p* = 0.629).

**Table 4:** MVPA attainment rates for young children of different sexes.

Classification	Boy (*n* = 70)	Girl (*n* = 79)	Total (*n* = 149)
Failure to attain the standards	12 (17.1%)	14 (17.7%)	26 (17.4%)
Attain the standards	58 (82 0.9%)	65 (82.3%)	123 (82.6%)

### 3.2 Levels of executive function in young children

The results of the young children’s executive function tests revealed that the score of inhibition was 43.58 ± 7.00, that of cognitive flexibility was 32.85 ± 5.78, that of working memory was 44.76 ± 7.36, and that of total executive function score was 104.32 ± 16.10 (Table 5).

**Table 5:** Young children’s executive functioning rating scale.

Classification	Inhibitory function	Cognitive flexibility	Working memory	Total executive function
Sex				
Boy (*n* = 70)	44.44 ± 7.24	33.23 ± 6.00	44.8 ± 7.82	105.09 ± 17.03
Girl (*n* = 79)	42.82 ± 6.72	32.51 ± 5.60	44.72 ± 6.98	103.63 ± 15.31
BMI rating				
Normal (*n* = 115)	43.26 ± 6.91	32.55 ± 5.76	44.71 ± 7.25	103.82 ± 16.00
Overweight (*n* = 19)	42.82 ± 6.21	33.00 ± 4.15	46.82 ± 7.31	106.27 ± 14.11
Obese (*n* = 15)	46.05 ± 7.75	34.58 ± 6.65	43.84 ± 8.22	106.32 ± 18.26
Average gross monthly income of parents (¥)				
1,000–5,000 (*n* = 44)	42.52 ± 6.37	32.86 ± 5.40	45.23 ± 7.79	103.34 ± 15.27
5,001–10,000 (*n* = 78)	43.68 ± 6.87	32.54 ± 5.56	44.13 ± 7.10	103.72 ± 15.61
>10,000 (*n* = 27)	45.04 ± 8.19	33.70 ± 7.03	45.81 ± 7.47	107.63 ± 18.83
Highest level of parental education				
Middle school and below (*n* = 48)	43.40 ± 7.03	33.15 ± 6.04	45.15 ± 7.13	104.87 ± 15.99
senior middle school (*n* = 52)	43.07 ± 7.71	32.24 ± 6.42	44.93 ± 7.32	104.00 ± 17.56
University and above (*n* = 47)	44.36 ± 6.11	33.23 ± 4.71	44.17 ± 7.74	104.11 ± 14.73
Total (*n* = 149)	43.58 ± 7.00	32.85 ± 5.78	44.76 ± 7.36	104.32 ± 16.10

### 3.3 Correlation analysis between physical activity and executive function in young children

The results of the correlation between physical activity and the demographic characteristics of young children revealed that LPA was positively correlated with the highest parental education level (*r* = 0.192, 95% CI: 0.030–0.354, *p* = 0.018) and BMI (*r* = 0.207, 95% CI: 0.048–0.366, *p* = 0.012), and that TPA was positively correlated with BMI (*r* = 0.169, 95% CI: 0.010–0.328, *p* = 0.036) (Table 6).

**Table 6:** Correlations between physical activity and demographic characteristics of young children.

Physical activity	Sex	BMI	Average gross monthly income of parents	Highest parental education
LPA	−0.089	0.207^*^	−0.071	0.192^*^
MVPA	−0.038	0.135	−0.078	0.119
TPA	−0.055	0.169^*^	−0.085	0.149

^*^p < 0.05.

The results of the correlation between physical activity and young children’s executive function revealed that MVPA was negatively correlated with inhibitory function (*r* = −0.226, 95% CI: −0.375 to −0.072, *p* = 0.005) and total executive function (*r* = −0.220, 95% CI: −0.367 to −0.063, *p* = 0.006), and also negatively correlated with cognitive flexibility (*r* = −0.206, 95% CI: −0.358 to −0.054, *p* = 0.008) and working memory (*r* = −0.208, 95% CI: −0.360 to −0.056, *p* = 0.007). Similarly, TPA was negatively correlated with inhibitory function (*r* = −0.215, 95% CI: −0.366 to −0.064, *p* = 0.006), and negatively correlated with cognitive flexibility (*r* = −0.193, 95% CI: −0.346 to −0.040, *p* = 0.012), working memory (*r* = −0.191, 95% CI: −0.344 to −0.038, *p* = 0.013), and total executive function (*r* = −0.207, 95% CI: −0.359 to −0.055, *p* = 0.008) (Table 7).

**Table 7:** Physical activity correlates with executive function in young children.

S. No	Variable	1	2	3	4	5	6	7
1	Inhibitory function	1						
2	Cognitive flexibility	0.883^**^	1					
3	Working memory	0.666^**^	0.640^**^	1				
4	Total executive function	0.912^**^	0.889^**^	0.890^**^	1			
5	LPA	−0.146	−0.134	−0.118	−0.141	1		
6	MVPA	−0.226^**^	−0.206^*^	−0.208^*^	−0.220^**^	0.684^**^	1	
7	TPA	−0.215^**^	−0.193^*^	−0.191^*^	−0.207^*^	0.837^**^	0.971^**^	1

^*^p < 0.05; ^**^p < 0.01.

### 3.4 Effect of physical activity on executive function in young children

#### 3.4.1 Relationships between physical activity and total executive function

The results of the linear regression analysis of the effect of physical activity on total executive function showed that in Model 1, MVPA (*β* = −0.211, standardized coefficient = −0.220, 95% CI: −0.364 to −0.059, *p* = 0.007) and TPA (*β* = −0.148, standardized coefficient = −0.207, 95% CI: −0.262 to −0.034, *p* = 0.011) were significantly correlated with total executive function, and the correlation of MVPA was greater (*p* < 0.01). In Model 2, MVPA (*β* = −0.223, standardized coefficient = −0.232, 95% CI: −0.377 to −0.069, *p* = 0.005) and TPA (*β* = −0.160, standardized coefficient = −0.223, 95% CI: −0.275 to −0.044, *p* = 0.007) were also significantly correlated with total executive function (*p* < 0.01). In Model 3, MVPA (*β* = −0.217, standardized coefficient = −0.226, 95% CI: −0.374 to −0.060, *p* = 0.007) and TPA (*β* = −0.156, standardized coefficient = −0.218, 95% CI: −0.274 to −0.037, *p* = 0.011) remained significantly correlated with total executive function (*p* < 0.05). This indicates that, within a certain range, for every 10-min/day increase in MVPA, the score of total executive function decreased by 2.17 points, and the effect of MVPA on total executive function was slightly stronger than that of TPA (*R*^2^: 0.034 > 0.029) (Table 8).

**Table 8:** Results of linear regression analysis of the effect of physical activity on executive function.

Model	Physical activity	*β* coefficient	Standardized coefficient	*p*-value	95% CI	*R* ^2^
Total executive function						
1	LPA	−0.302	−0.141	0.086	−0.647 to 0.043	0.013
	MVPA	−0.211	−0.220	0.007	−0.364 to −0.059	0.042
	TPA	−0.148	−0.207	0.011	−0.262 to −0.034	0.036
2	LPA	−0.343	−0.160	0.056	−0.696 to 0.010	0.015
	MVPA	−0.223	−0.232	0.005	−0.377 to −0.069	0.044
	TPA	−0.160	−0.223	0.007	−0.275 to −0.044	0.039
3	LPA	−0.328	−0.153	0.078	−0.693 to 0.037	0.006
	MVPA	−0.217	−0.226	0.007	−0.374 to −0.060	0.034
	TPA	−0.156	−0.218	0.011	−0.274 to −0.037	0.029
Inhibitory function						
1	LPA	−0.135	−0.146	0.076	−0.285 to 0.015	0.015
	MVPA	−0.094	−0.226	0.006	−0.160 to −0.028	0.045
	TPA	−0.067	−0.215	0.008	−0.116 to −0.017	0.040
2	LPA	−0.165	−0.178	0.033	−0.317 to −0.013	0.031
	MVPA	−0.103	−0.247	0.003	−0.169 to −0.037	0.061
	TPA	−0.075	−0.242	0.003	−0.125 to −0.025	0.058
3	LPA	−0.172	−0.185	0.031	−0.328 to −0.016	0.033
	MVPA	−0.103	−0.248	0.003	−0.170 to −0.036	0.062
	TPA	−0.076	−0.246	0.003	−0.127 to −0.026	0.060
Cognitive flexibility						
1	LPA	−0.103	−0.134	0.102	−0.227 to −0.021	0.011
	MPA	−0.131	−0.194	0.018	−0.238 to −0.023	0.031
	TPA	−0.049	−0.193	0.018	−0.091 to −0.008	0.031
2	LPA	−0.127	−0.165	0.048	−0.253 to −0.001	0.026
	MVPA	−0.078	−0.226	0.006	−0.133 to −0.023	0.050
	TPA	−0.056	−0.218	0.008	−0.097 to −0.015	0.046
3	LPA	−0.132	−0.172	0.048	−0.262 to −0.001	0.014
	MVPA	−0.079	−0.229	0.006	−0.135 to −0.023	0.038
	TPA	−0.058	−0.224	0.008	−0.100 to −0.015	0.035
Working memory						
1	LPA	−0.116	−0.118	0.150	−0.274 to 0.042	0.007
	MVPA	−0.091	−0.208	0.011	−0.161 to −0.021	0.037
	TPA	−0.062	−0.191	0.019	−0.115 to −0.010	0.030
2	LPA	−0.117	−0.120	0.155	−0.280 to 0.045	0.001
	MVPA	−0.092	−0.210	0.011	−0.163 to −0.021	0.030
	TPA	−0.063	−0.194	0.020	−0.117 to −0.010	0.024
3	LPA	−0.110	−0.112	0.199	−0.278 to 0.058	0.012
	MVPA	−0.090	−0.206	0.015	−0.163 to −0.018	0.018
	TPA	−0.062	−0.190	0.026	−0.117 to −0.007	0.011

Model 1: uncontrolled; Model 2: controlled for BMI; Model 3: controlled for BMI, highest parental education, and average gross monthly parental income; LPA, light physical activity; MVPA, medium to high physical activity; TPA, total physical activity.

**Figure 1:**
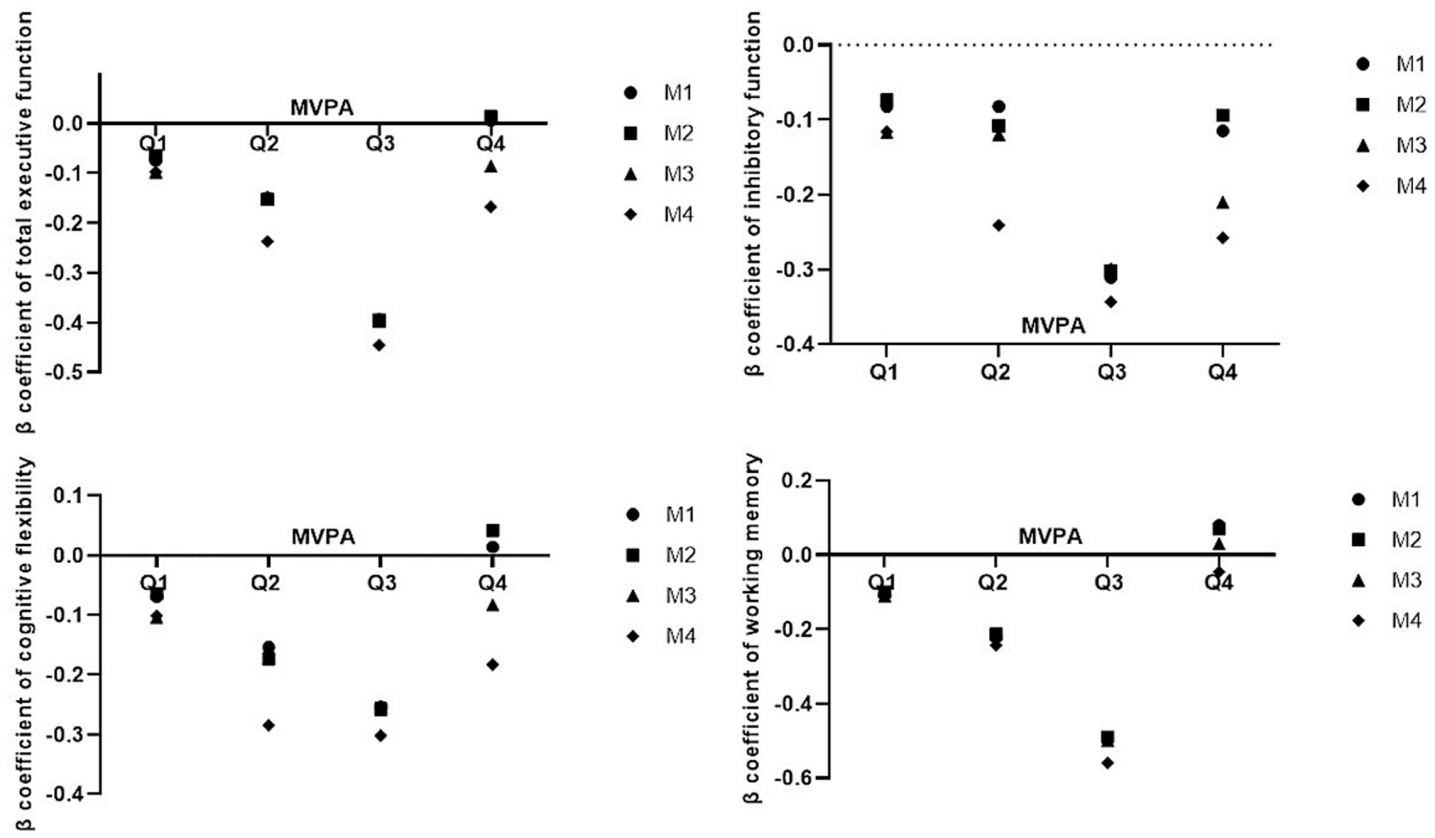
Dose effect relationship between MVPA and executive function scores.

The overall results of MVPA were divided into four groups according to interquartile spacing, and linear regression modeling revealed a “U”-shaped relationship between MVPA and total executive function scores, whereas total executive function scores were negatively correlated with total executive function performance. That is, there was an inverted “U”-shaped dose–effect relationship between MVPA and total executive function performance. Among the four models, the absolute value of the *β* coefficient of the effect of MVPA on total executive function in group Q3 (71.39 min/day ≤ MVPA < 83.33 min/day) was greater than that in groups Q1 (MVPA < 64.04 min/day), Q2 (64.04 min/day ≤ MVPA < 71.39 min/day), and Q4 (MVPA ≥ 83.33 min/day) (Table 9; Figure 1). These findings suggested that MVPA from 71.39 min/day to 83.33 min/day resulted in the greatest improvement in total executive function.

**Table 9:** β coefficient (95% CI) for the effect of MVPA on executive function.

Model	Q1	Q2	Q3	Q4
Total executive function				
M1	−0.074 (−1.314 to 0.841)	−0.150 (−3.411 to 1.315)	−0.393* (−2.622 to −0.287)	0.006 (−0.357 to 0.369)
M2	−0.065 (−1.281 to 0.866)	−0.152 (−3.482 to 1.366)	−0.395* (−2.664 to −0.258)	0.014 (−0.361 to 0.391)
M3	−0.098 (−1.399 to 0.772)	−0.147 (−3.542 to 1.491)	−0.398* (−2.701 to −0.243)	−0.085 (−0.464 to 0.283)
M4	−0.097 (−1.430 to 0.810)	−0.237 (−4.247 to 0.952)	−0.445* (−2.924 to −0.367)	−0.167 (−0.556 to −0.203)
Inhibitory function				
M1	−0.082 (−0.552 to 0.335)	−0.082 (−1.243 to 0.761)	−0.311 (−1.109 to 0.025)	−0.115 (−0.226 to 0.112)
M2	−0.073 (−0.537 to 0.345)	−0.108 (−1.327 to 0.694)	−0.302 (−1.108 to 0.058)	−0.094 (−0.221 to 0.128)
M3	−0.117 (−0.594 to 0.285)	−0.120 (−1.400 to 0.696)	−0.299 (−1.115 to 0.076)	−0.210 (−0.273 to 0.064)
M4	−0.116 (−0.603 to 0.298)	−0.241 (−1.741 to 0.329)	−0.343 (−1.208 to 0.015)	−0.258 (−0.303 to 0.047)
Cognitive flexibility				
M1	−0.069 (−0.496 to 0.327)	−0.154 (−0.980 to 0.368)	−0.254 (−0.846 to 0.112)	0.014 (−0.134 to 0.146)
M2	−0.064 (−0.493 to −0.337)	−0.173 (−1.028 to 0.343)	−0.257 (−0.865 to 0.122)	0.042 (−0.127 to 0.161)
M3	−0.104 (−0.543 to 0.290)	−0.173 (−1.056 to 0.367)	−0.259 (−0.878 to 0.130)	−0.083 (−0.171 to 0.103)
M4	−0.101 (−0.554 to 0.306)	−0.285 (−1.263 to 0.130)	−0.302 (−0.963 to 0.092)	−0.183 (−0.209 to 0.060)
Working memory				
M1	−0.108 (−0.608 to 0.313)	−0.222 (−2.188 to 0.440)	−0.491** (−1.118 to −0.271)	0.080 (−0.132 to 0.213)
M2	−0.099 (−0.595 to 0.323)	−0.213 (−2.183 to 0.508)	−0.490** (−1.129 to −0.257)	0.070 (−0.144 to 0.214)
M3	−0.111 (−0.624 to 0.321)	−0.212 (−2.231 to 0.563)	−0.499** (−1.149 to −0.262)	0.031 (−0.171 to 0.203)
M4	−0.111 (−0.641 to 0.336)	−0.243 (−2.450 to 0.542)	−0.559** (−1.249 to −0.333)	−0.046 (−0.217 to 0.170)

M1: uncontrolled; M2: controlled for sex; M3: controlled for sex, BMI; M4: controlled for sex, BMI, highest parental education, and average gross monthly parental income. Quartiles of mean daily MVPA time (min/day): Q1: MVPA < 64.04; Q2: 64.04 ≤ MVPA < 71.39; Q3: 71.39 ≤ MVPA < 83.33; Q4: MVPA ≥ 83.33; ^*^p < 0.05.

The results of the linear regression analysis of the effect of physical activity on boys’ total executive function showed that in Model 1, MVPA (*β* = −0.311, standardized coefficient = −0.304, 95% CI: −0.547 to −0.075, *p* = 0.010) and TPA (*β* = −0.219, standardized coefficient = −0.289, 95% CI: −0.395 to −0.044, *p* = 0.015) were significantly correlated with boys’ total executive function (*p* < 0.05). In Model 2, MVPA (*β* = −0.326, standardized coefficient = −0.319, 95% CI: −0.563 to −0.090, *p* = 0.007) and TPA (*β* = −0.241, standardized coefficient = −0.317, 95% CI: −0.417 to −0.064, *p* = 0.008) remained significantly correlated with total executive function (*p* < 0.01). In Model 3, MVPA (*β* = −0.315, standardized coefficient = −0.308, 95% CI: −0.560 to −0.070, *p* = 0.013) and TPA (*β* = −0.230, standardized coefficient = −0.304, 95% CI: −0.415 to −0.046, *p* = 0.015) were still significantly correlated with boys’ total executive function (*p* < 0.05). This indicates that, within a certain range, for every 10 min/day increase in MVPA, the score of total executive function decreased by 3.15 points, and the effect of MVPA on total executive function was slightly stronger than that of TPA (*R*^2^: 0.064 > 0.059) (Table 10). In contrast, no significant correlation was found between physical activity and total executive function among girls.

**Table 10:** Results of linear regression analysis of the effect of physical activity on executive function in boys.

Model	Physical activity	*β* coefficient	Standardized coefficient	*p*-value	95% CI	*R* ^2^
Total executive function						
1	LPA	−0.406	−0.185	0.125	−0.927 to 0.115	0.020
	MVPA	−0.311	−0.304	0.010	−0.547 to −0.075	0.079
	TPA	−0.219	−0.289	0.015	−0.395 to −0.044	0.070
2	LPA	−0.467	−0.213	0.102	−1.028 to 0.095	0.011
	MVPA	−0.326	−0.319	0.007	−0.563 to −0.090	0.087
	TPA	−0.241	−0.317	0.008	−0.417 to −0.064	0.084
3	LPA	−0.467	−0.213	0.102	−1.028 to 0.095	0.011
	MVPA	−0.315	−0.308	0.013	−0.560 to −0.070	0.064
	TPA	−0.230	−0.304	0.015	−0.415 to −0.046	0.059
Inhibitory function						
1	LPA	−0.155	−0.167	0.168	−0.377 to 0.067	0.013
	MVPA	−0.142	−0.326	0.006	−0.241 to −0.042	0.093
	TPA	−0.096	−0.299	0.012	−0.171 to −0.022	0.076
2	LPA	−0.222	−0.239	0.052	−0.447 to 0.002	0.068
	MVPA	−0.153	−0.352	0.003	−0.250 to −0.055	0.139
	TPA	−0.111	−0.344	0.004	−0.184 to −0.038	0.132
3	LPA	−0.225	−0.241	0.059	−0.458 to 0.008	0.056
	MVPA	−0.148	−0.341	0.005	−0.250 to −0.047	0.119
	TPA	−0.109	−0.338	0.006	−0.185 to −0.033	0.114
Cognitive flexibility						
1	LPA	−0.076	−0.098	0.418	−0.262 to 0.110	0.005
	MVPA	−0.095	−0.265	0.027	−0.179 to −0.011	0.057
	TPA	−0.061	−0.230	0.055	−0.124 to 0.001	0.039
2	LPA	−0.131	−0.169	0.170	−0.318 to 0.057	0.047
	MVPA	−0.105	−0.291	0.013	−0.187 to −0.023	0.106
	TPA	−0.074	−0.276	0.020	−0.135 to −0.012	0.096
3	LPA	−0.123	−0.159	0.215	−0.319 to 0.073	0.030
	MVPA	−0.100	−0.277	0.023	−0.185 to −0.014	0.050
	TPA	−0.070	−0.263	0.034	−0.135 to −0.006	0.073
Working memory						
1	LPA	−0.205	−0.204	0.091	−0.443 to 0.033	0.027
	MVPA	−0.131	−0.280	0.019	−0.241 to −0.022	0.065
	TPA	−0.097	−0.278	0.020	−0.178 to −0.016	0.064
2	LPA	−0.222	−0.220	0.080	−0.470 to 0.027	0.016
	MVPA	−0.133	−0.283	0.019	−0.244 to −0.023	0.052
	TPA	−0.100	−0.287	0.019	−0.182 to −0.017	0.052
3	LPA	−0.208	−0.206	0.115	−0.467 to 0.052	0.004
	MVPA	−0.138	−0.293	0.019	−0.252 to −0.024	0.042
	TPA	−0.101	−0.290	0.022	−0.187 to −0.015	0.039

Model 1: uncontrolled; Model 2: controlled for BMI; Model 3: controlled for BMI, highest parental education, and average gross monthly parental income; LPA, light physical activity; MVPA, medium to high physical activity; TPA, total physical activity.

#### 3.4.2 Relationship between physical activity and inhibitory function

The results of the linear regression analysis of the effect of physical activity on inhibitory function showed that in Model 1, MVPA (*β* = −0.094, standardized coefficient = −0.226, 95% CI: −0.160 to −0.028, *p* = 0.006) and TPA (β = −0.067, standardized coefficient = −0.215, 95% CI: −0.116 to −0.017, *p* = 0.008) were significantly correlated with inhibitory function (*p* < 0.01). In Model 2, LPA (*β* = −0.165, standardized coefficient = −0.178, 95% CI: −0.317 to −0.013, *p* = 0.033), MVPA (*β* = −0.103, standardized coefficient = −0.247, 95% CI: −0.169 to −0.037, *p* = 0.003), and TPA (*β* = −0.075, standardized coefficient = −0.242, 95% CI: −0.125 to −0.025, *p* = 0.003) were all significantly correlated with inhibitory function (*p* < 0.05), with stronger correlations for MVPA and TPA (*p* < 0.01). In Model 3, LPA (*β* = −0.172, standardized coefficient = −0.185, 95% CI: −0.328 to −0.016, *p* = 0.031), MVPA (*β* = −0.103, standardized coefficient = −0.248, 95% CI: −0.170 to −0.036, *p* = 0.003), and TPA (*β* = −0.076, standardized coefficient = −0.246, 95% CI: −0.127 to −0.026, *p* = 0.003) remained significantly correlated with inhibitory function (*p* < 0.05), and the correlations of MVPA and TPA were stronger (*p* < 0.01). This indicates that, within a certain range, for every 10-min/day increase in MVPA, the inhibitory function score decreased by 1.03 points (95% CI: −0.170 to −0.036), and the effect of MVPA on inhibitory function was slightly stronger than that of TPA (*R*^2^: 0.062 > 0.060) (Table 8).

The overall results of MVPA were divided into four groups according to interquartile spacing, and linear regression analysis revealed a “U”-shaped relationship between MVPA and inhibitory function scores, whereas inhibitory function scores were negatively correlated with inhibitory function performance. In other words, there was an inverted “U” shaped dose effect relationship between MVPA and inhibitory performance (Table 9, Figure 1).

The results of the boys’ analysis revealed that in Model 1, MVPA (*β* = −0.142, standardized coefficient = −0.326, 95% CI: −0.241 to −0.042, *p* = 0.006) and TPA (*β* = −0.096, standardized coefficient = −0.299, 95% CI: −0.171 to −0.022, *p* = 0.012) were significantly correlated with inhibitory function (*p* < 0.05), with a stronger correlation observed for MVPA (*p* < 0.01). In Model 2, MVPA (*β* = −0.153, standardized coefficient = −0.352, 95% CI: −0.250 to −0.055, *p* = 0.003) and TPA (*β* = −0.111, standardized coefficient = −0.344, 95% CI: −0.184 to −0.038, *p* = 0.004) remained significantly correlated with inhibitory function (*p* < 0.01). In Model 3, MVPA (*β* = −0.148, standardized coefficient = −0.341, 95% CI: −0.250 to −0.047, *p* = 0.005) and TPA (*β* = −0.109, standardized coefficient = −0.338, 95% CI: −0.185 to −0.033, *p* = 0.006) were still significantly correlated with inhibitory function (*p* < 0.01). This indicates that, within a certain range, for every 10-min/day increase in MVPA, the inhibitory function score decreased by 1.48 points (95% CI: −0.250 to −0.047), and the effect of MVPA on inhibitory function was slightly stronger than that of TPA (*R*^2^: 0.119 > 0.114) (Table 10). In contrast, no significant correlation was found between physical activity and inhibitory function among girls.

#### 3.4.3 Relationship between physical activity and cognitive flexibility

The results of the linear regression analysis of the effect of physical activity on cognitive flexibility revealed that in Model 1, MVPA (*β* = −0.131, standardized coefficient = −0.194, 95% CI: −0.238 to −0.023, *p* = 0.018) and TPA (*β* = −0.049, standardized coefficient = −0.193, 95% CI: −0.091 to −0.008, *p* = 0.018) were significantly correlated with cognitive flexibility (*p* < 0.05). In Model 2, LPA (*β* = −0.127, standardized coefficient = −0.165, 95% CI: −0.253 to −0.001, *p* = 0.048), MVPA (*β* = −0.078, standardized coefficient = −0.226, 95% CI: −0.133 to −0.023, *p* = 0.006), and TPA (*β* = −0.056, standardized coefficient = −0.218, 95% CI: −0.097 to −0.015, *p* = 0.008) were all significantly correlated with cognitive flexibility (*p* < 0.05), with stronger correlations observed for MVPA and TPA (*p* < 0.01). In Model 3, LPA (*β* = −0.132, standardized coefficient = −0.172, 95% CI: −0.262 to −0.001, *p* = 0.048), MVPA (*β* = −0.079, standardized coefficient = −0.229, 95% CI: −0.135 to −0.023, *p* = 0.006), and TPA (*β* = −0.058, standardized coefficient = −0.224, 95% CI: −0.100 to −0.015, *p* = 0.008) remained significantly correlated with cognitive flexibility (*p* < 0.05), and the correlations for MVPA and TPA were higher (*p* < 0.01). This indicates that, within a certain range, for every 10 min/day increase in MVPA, the cognitive flexibility score decreased by 0.79 points (95% CI: −0.135 to −0.023), and the effect of MVPA on cognitive flexibility was slightly stronger than that of TPA (*R*^2^: 0.038 > 0.035) (Table 8).

The overall results of MVPA were divided into four groups according to interquartile spacing, and linear regression analysis revealed a “U”-shaped relationship between MVPA and cognitive flexibility scores, whereas cognitive flexibility scores were negatively correlated with cognitive flexibility performance. In other words, there was an inverted “U” shaped dose effect relationship between MVPA and cognitive flexibility performance (Table 9, Figure 1).

The results of the boys’ analyses showed that in Model 1, MVPA (*β* = −0.095, standardized coefficient = −0.265, 95% CI: −0.179 to −0.011, *p* = 0.027) was significantly correlated with cognitive flexibility (*p* < 0.05). In Model 2, MVPA (*β* = −0.105, standardized coefficient = −0.291, 95% CI: −0.187 to −0.023, *p* = 0.013) and TPA (*β* = −0.074, standardized coefficient = −0.276, 95% CI: −0.135 to −0.012, *p* = 0.020) were significantly correlated with cognitive flexibility (*p* < 0.05). In Model 3, MVPA (*β* = −0.100, standardized coefficient = −0.277, 95% CI: −0.185 to −0.014, *p* = 0.023) and TPA (*β* = −0.070, standardized coefficient = −0.263, 95% CI: −0.135 to −0.006, *p* = 0.034) remained significantly correlated with cognitive flexibility (*p* < 0.05). This indicates that, within a certain range, for every 10 min/day increase in TPA, the cognitive flexibility score decreased by 0.70 points (95% CI: −0.135 to −0.006), and the effect of TPA on cognitive flexibility was slightly stronger than that of MVPA (*R*^2^: 0.073 > 0.050) (Table 10). In contrast, no significant correlation was found between physical activity and cognitive flexibility among girls.

#### 3.4.4 Relationship between physical activity and working memory

The results of the linear regression analysis of the effect of physical activity on working memory showed that in Model 1, MVPA (*β* = −0.091, standardized coefficient = −0.208, 95% CI: −0.161 to −0.021, *p* = 0.011) and TPA (*β* = −0.062, standardized coefficient = −0.191, 95% CI: −0.115 to −0.010, *p* = 0.019) were significantly correlated with working memory (*p* < 0.05). In Model 2, MVPA (*β* = −0.092, standardized coefficient = −0.210, 95% CI: −0.163 to −0.021, *p* = 0.011) and TPA (*β* = −0.063, standardized coefficient = −0.194, 95% CI: −0.117 to −0.010, *p* = 0.020) remained significantly correlated with working memory (*p* < 0.05). In Model 3, MVPA (*β* = −0.090, standardized coefficient = −0.206, 95% CI: −0.163 to −0.018, *p* = 0.015) and TPA (*β* = −0.062, standardized coefficient = −0.190, 95% CI: −0.117 to −0.007, *p* = 0.026) continued to show significant correlations with working memory (*p* < 0.05). This indicates that, within a certain range, for every 10 min/day increase in MVPA, the working memory score decreased by 0.90 points (95% CI: −0.163 to −0.018), and the effect of MVPA on working memory was slightly stronger than that of TPA in older children (*R*^2^: 0.018 > 0.011) (Table 8).

The overall results of MVPA were divided into four groups according to interquartile spacing, and the results of linear regression modeling revealed a “U”-shaped relationship between MVPA and working memory scores, whereas working memory scores were negatively correlated with working memory performance. In other words, there was an inverted “U”-shaped dose–effect relationship between MVPA and working memory performance. Among the four models, the absolute value of the β coefficient of the effect of MVPA on young children’s working memory was greater in group Q3 (71.39 min/day ≤ MVPA < 83.33 min/day) than in groups Q1 (MVPA < 64.04 min/day), Q2 (64.04 min/day ≤ MVPA < 71.39 min/day), and Q4 (MVPA ≥ 83.33 min/day). It is suggested that MVPA from 71.39 min/day to 83.33 min/day has the greatest effect on improving working memory (Table 9, Figure 1).

The results of the boys’ analyses showed that in Model 1, MVPA (*β* = −0.131, standardized coefficient = −0.280, 95% CI: −0.241 to −0.022, *p* = 0.019) and TPA (*β* = −0.097, standardized coefficient = −0.278, 95% CI: −0.178 to −0.016, *p* = 0.020) were significantly correlated with working memory (*p* < 0.05). In Model 2, MVPA (*β* = −0.133, standardized coefficient = −0.283, 95% CI: −0.244 to −0.023, *p* = 0.019) and TPA (*β* = −0.100, standardized coefficient = −0.287, 95% CI: −0.182 to −0.017, *p* = 0.019) remained significantly correlated with working memory (*p* < 0.05). In Model 3, MVPA (*β* = −0.138, standardized coefficient = −0.293, 95% CI: −0.252 to −0.024, *p* = 0.019) and TPA (*β* = −0.101, standardized coefficient = −0.290, 95% CI: −0.187 to −0.015, *p* = 0.022) continued to show significant correlations with working memory (*p* < 0.05). This indicates that, within a certain range, for every 10 min/day increase in MVPA, the working memory score decreased by 1.38 points (95% CI: −0.252 to −0.024), and the effect of MVPA on working memory was slightly stronger than that of TPA (*R*^2^: 0.049 > 0.039) (Table 10). In contrast, no significant correlation was found between physical activity and working memory among girls.

## 4 Discussion

### 4.1 Analysis of the physical activity levels of young children

The beneficial effects of physical activity on health at different stages of an individual’s life have been widely documented (Guan et al., 2020). In line with this background, this section analyzes the objectively measured physical activity levels among 5–6-year-old preschoolers and compares them with international guidelines. Currently, the physical activity of young children is measured via a parent-administered physical activity questionnaire and an accelerometer. Accelerometer measurements are more objective, but different settings of the measurement parameters can lead to large gaps in the measurement results, in which the choice of intensity thresholds is crucial (Dupuy and Tremblay, 2019). In this study, a 1-s sampling interval and Pate cut-off points were used, which differ from the longer epoch lengths adopted in several previous studies and may have influenced comparability of LPA and TPA estimates. According to the results of several published surveys that used Pate intensity thresholds to test physical activity in 3–6-year-old toddlers, the MVPA time range was 33.5–99.0 min/day (Hoffmann and Powlishta, 2001). Ltifi et al. (2024a) and Ltifi et al. (2024c) also reported similar patterns in preschool cohorts. In contrast, the findings of the present study revealed that the MVPA time for 5–6-year-olds was 74.55 ± 16.77 min/day, which is in the middle of the published data.

A comparison of physical activity levels between boys and girls revealed that there was no difference between their physical activities, but boys’ LPA, MVPA, and TPA times were greater than those of girls, which was consistent with the results of previous studies. This may be related to gender-related play preferences, differences in parental encouragement, or boys’ greater involvement in outdoor free-play behaviors (Calcaterra et al., 2022). In this study, we graded the BMI of young children according to the BMI growth curve of Chinese children and adolescents aged 0–18 years (Li, 2023) and reported that LPA and TPA were correlated with overweight young children (LPA: *r* = 0.207, *p* = 0.012; TPA: *r* = 0.169, *p* = 0.036) and that LPA was correlated with obese young children (*r* = 0.207, *p* = 0.012), which may be related to the lower proportion of overweight and obese individuals in the investigated population.

With respect to the duration and intensity of exercise for young children, the World Health Organization (WHO), a few developed countries and China have issued physical activity guidelines for young children. The guidelines generally recommend that the total daily exercise time for young children should not be less than 180 min, of which medium- and higher-intensity exercise should last no less than 60 min (Guan et al., 2020). However, in the findings of this study, the average TPA time of young children was 110.84 ± 22.52 min/day, the MVPA time was 74.55 ± 16.77 min/day, and 82.6% of the young children achieved the recommended amount of MVPA but not TPA. Environmental and contextual factors, such as limited outdoor play space in kindergartens and structured daily schedules that reduce free-play duration, may partly explain lower TPA despite adequate MVPA. We speculate that this may also be due to the setting of the accelerometer acquisition interval. Because physical activity in preschool children is characterized by suddenness, a fast frequency of intensity change and a short duration, the sampling interval should be as short as possible when measuring physical activity in preschool children via accelerometers. Excessively long sampling intervals may result in an overrepresentation of LPA in the measurements, while the impact on MVPA is relatively minor across different sampling intervals (Dupuy and Tremblay, 2019). In this study, to measure the physical activity level of preschool children more accurately, a sampling interval of 1 s was used, which resulted in an LPA result of only 36.23 min/day and less TPA time. Existing methodological studies have supported the use of 1-s epochs to better capture intermittent preschooler movement patterns (Ltifi et al., 2024a). Since medium- and high-intensity physical activity is more beneficial to physical and mental health, researchers have focused more on medium- and high-intensity physical activity surveys.

This study was based on objective measurements of physical activity in young children, and the findings revealed that the level of daily physical activity (MVPA = 74.55 ± 16.77 min/day) in 5–6-year-olds was in the middle of the range compared with data from similar studies. According to the recommended amount of physical activity guidelines for young children (Guan et al., 2020), 17.4% of the young children tested did not perform MVPA. In terms of TPA, physical activity in young children should be increased. This suggests that more attention should be given to the duration of physical activity in young children in future studies, and intervention strategies such as increasing outdoor free-play sessions and embedding active routines into classroom activities may help promote overall activity duration.

### 4.2 Effects of physical activity on executive function

With respect to research on the effects of physical activity on executive function, neurophysiological theories suggest an inverted “U” relationship between exercise intensity and executive function performance, with the stress generated by high-load exercise contributing to an increase in dopamine and norepinephrine secretion in the prefrontal cortex, leading to decreased neuronal firing and impaired high-level cognitive processing. In contrast, low- to medium-load exercise produces moderate stress, which promotes neuronal activity and enhances executive function (Quan et al., 2020). This theoretical framework has been well supported by neurophysiological evidence, including the meta-analysis by McMorris and Hale (2012), which demonstrated a curvilinear relationship between acute exercise intensity and cognitive performance. Consistent with these mechanisms, the current study found an inverted “U”-shaped association between MVPA and executive function.

Moreover, studies on the effects of physical activity interventions on executive function in children aged 3–7 years have shown positive effects on inhibitory function, cognitive flexibility, and working memory (Li et al., 2020). However, some studies have also shown that physical activity is beneficial only for inhibitory functioning but not for cognitive flexibility (Álvarez-Bueno et al., 2017), and MVPA in young children is more strongly associated with inhibitory functioning dimensions (Chang and Wang, 2020). Physical activity has also been shown to be unrelated to inhibitory functioning and cognitive flexibility in young children but is correlated with working memory (Cook et al., 2019). These inconsistencies may be related to differences in physical activity intensity cut-off points and variations in executive function assessment tools. In this regard, in the experiment, we chose the Executive Function Measurement Scale, which focuses on the daily performance of young children’s executive function. While parent-rated questionnaires such as the BRIEF-P can comprehensively reflect children’s daily behaviors, they may also introduce reporting bias, combining them with objective tests may improve accuracy in future studies. Moreover, the subject groups in previous studies often had a wider age span, and important developmental changes in executive functioning occur at the age of 2–5 years. In contrast, this study focused on 5–6-year-olds, whose developmental variability is relatively stable, thereby enhancing internal validity. The present study found an inverted “U”-shaped dose–effect relationship between MVPA and executive function and its subdomains, further supporting the potential benefits of moderate MVPA for preschool children.

In terms of the effect of physical activity on total executive function, the higher the total executive function score is, the more severe the impairment of total executive function. In this study, after correcting for sociological influences in the linear regression analysis, MVPA showed a significant negative association with total executive function (*β* = −0.217, *p* = 0.007, 95% CI: −0.374 to −0.060, *R*^2^ = 0.034), and MVPA was more effective than TPA in improving total executive function. Moreover, in previous studies on the correlation between physical activity and executive function subdomains, the effect of physical activity on total executive function has been less frequently studied, but intervention experiments such as soccer games, “group rule games,” and tennis games have promoted the overall executive function performance of young children (Wang et al., 2018; Xu et al., 2022; Kou et al., 2024). The inverted “U”-shaped dose–effect relationship observed in this study is consistent with neurophysiological theories of exercise intensity and executive functioning. Among them, MVPA between 71.39 and 83.33 min/day produced the greatest improvement in total executive function. The findings suggest that moderate MVPA can be added to children’s daily routines in preschool or in therapeutic settings. Teachers might include short bouts of moderate activity at suitable points in the day or add short bursts of movement between learning tasks. Such approaches may help support children with executive function difficulties. This provides a practical basis for using physical activity to improve executive functioning in children with developmental disorders.

With respect to the effects of physical activity on inhibition, this study showed that LPA, MVPA, and TPA were all correlated with inhibitory performance, with MVPA showing the strongest association and a clear inverted “U”-shaped dose–effect pattern. Previous studies similarly reported that moderate-intensity physical activity significantly improves inhibition in preschoolers. For example, long-term soccer or tennis activities enhanced frontal lobe activation and improved inhibitory control in 5–6-year-old children (Jiang and Zeng, 2015; Xu et al., 2022; Esteban-Cornejo et al., 2014). Based on these findings, physical activity appears to have a clear positive effect on improving inhibitory function, MVPA appears to exert the most notable benefit on inhibitory function.

With respect to cognitive flexibility, this study found that LPA, MVPA, and TPA were correlated with flexibility, with MVPA showing relatively stronger effects and an inverted “U”-shaped pattern. Previous studies, however, have reported inconsistent results, with some showing effects only on inhibition (Chang and Wang, 2020) and others finding no association with flexibility (Killgore and Schwab, 2012). These differences may relate to variations in intervention type, training duration, task demands, or cultural and environmental contexts. For instance, movement behaviors differ substantially across preschool settings, as demonstrated in Ltifis’ et al., 2024b findings on urban–rural differences in children’s activity patterns. Considering these factors, the present results suggest that MVPA may play a meaningful role in promoting cognitive flexibility in 5–6-year-olds.

With respect to working memory, this study found that MVPA and TPA were correlated with working-memory performance, with MVPA showing a slightly greater effect. The quartile-based regression confirmed an inverted “U”-shaped relationship, with 71.39–83.33 min/day of MVPA producing the most pronounced improvements. Previous studies also reported similar associations (Cook et al., 2019; Xu et al., 2022). These benefits may be partly explained by increased activation of the prefrontal cortex, enhanced cerebral blood flow, and improved synaptic plasticity, which support the neural processes underlying working-memory performance (Li, 2023). Overall, these findings provide support for using moderate MVPA to enhance working memory in young children.

### 4.3 Sex differences in the effect of physical activity on executive function

In terms of sex differences in the effects of physical activity on executive function, we found that there was a correlation between physical activity and executive function in boys but not in girls. In a study related to the effect of physical activity on cognitive ability in preschoolers, the same correlation between physical activity level and cognitive ability was found to exist only in males (Quan et al., 2020). However, it was also found that the amount of daily physical activity was correlated with IQ in females but not males (Killgore and Schwab, 2012) and that MVPA was positively correlated with academic achievement in females (Visier-Alfonso et al., 2021).

Although the underlying causes of the sex differences in the effect of physical activity on executive function remain unclear, existing studies (Huang et al., 2022) and our findings suggest several possible explanations: (1) differences in the baseline level of cardiorespiratory endurance and cognitive ability, (2) differences in physiological responses, and (3) differences in psychological responses. The reasons for the sex differences in the effects of physical activity on executive function in young children are inconclusive and need to be further explored in subsequent studies. Moreover, the current status of physical activity among girls should receive more attention.

## 5 Conclusion

Physical activity improved executive function to some extent in 5–6-year-old young children. Among them, MVPA improved executive function and subdomain scores in young children, while there was a correlation between physical activity and executive function in boys.

## Data Availability

The raw data supporting the conclusions of this article will be made available by the authors, without undue reservation.
